# A Missile-Borne Angular Velocity Sensor Based on Triaxial Electromagnetic Induction Coils

**DOI:** 10.3390/s16101625

**Published:** 2016-09-30

**Authors:** Jian Li, Dan Wu, Yan Han

**Affiliations:** Institute of Signal Capturing & Processing Technology, Key Laboratory of Shanxi Province, North University of China, Taiyuan 030051, China; wudan910201@126.com (D.W.); hanyan@nuc.edu.cn (Y.H.)

**Keywords:** angular velocity, angular displacement, self-adaptive frequency tracking measurement, FPGA

## Abstract

Aiming to solve the problem of the limited measuring range for angular motion parameters of high-speed rotating projectiles in the field of guidance and control, a self-adaptive measurement method for angular motion parameters based on the electromagnetic induction principle is proposed. First, a framework with type bent “I-shape” is used to design triaxial coils in a mutually orthogonal way. Under the condition of high rotational speed of a projectile, the induction signal of the projectile moving across a geomagnetic field is acquired by using coils. Second, the frequency of the pulse signal is adjusted self-adaptively. Angular velocity and angular displacement are calculated in the form of periodic pulse counting and pulse accumulation, respectively. Finally, on the basis of that principle prototype of the sensor is researched and developed, performance of measuring angular motion parameters are tested on the sensor by semi-physical and physical simulation experiments, respectively. Experimental results demonstrate that the sensor has a wide measuring range of angular velocity from 1 rps to 100 rps with a measurement error of less than 0.3%, and the angular displacement measurement error is lower than 0.2°. The proposed method satisfies measurement requirements for high-speed rotating projectiles with an extremely high dynamic range of rotational speed and high precision, and has definite value to engineering applications in the fields of attitude determination and geomagnetic navigation.

## 1. Introduction

High-speed rotating projectiles, with rotational speeds generally in a range from 2 rps to 50 rps, have become one of the most important kinds of ammunition among conventional ammunition. On the one hand, a high-speed rotating projectile ensures flight stability by way of rotation; on the other hand, this kind of projectile changes flight attitude and corrects its flight trajectory through adjusting its rotational speed, so as to accurately attack targets. In the course of structure improvement and performance testing of high-speed rotating projectiles, the accurate measurement of angular motion parameters, such as angular velocity and angular displacement, is key for optimizing the structure and improve the attacking accuracy. The precision guidance of high-speed rotating projectiles is a research hotspot in the field of international arms and ammunition at present.

Scientific researchers mainly use gyroscopes to obtain the angular velocity information of projectiles nowadays [1,2,3,4,5,6,7,8,9,10,11,12]. For example, the angular velocity sensor based on a magnetically suspended control moment gyroscope (MSCMG) [13], the capacitive angular velocity sensor and the angular velocity sensor based on magnetohydrodynamics at low frequency [14,15], etc. Although these sensors have certain advantages of small volume and high damage impact resistance, they still have problems of small measuring ranges, zero drift and high cumulative errors when measuring rotational speed. Though some of the errors can be corrected by some algorithms like Kalman prediction, the requirements of large dynamic range and high precision for measuring the rotational speed of high-speed rotating projectiles still cannot be satisfied under actual combat conditions. Aiming at addressing the problems described above, this paper presents a method for measuring angular motion parameters based on the electromagnetic induction principle. The specific working of this method is as follows: under different rotation speeds, an induction coil of a bent “I-shape” type is used to acquire signals of a high-speed rotating projectile moving across a geomagnetic field. A self-adaptive frequency tracking measurement algorithm is proposed to obtain the angular velocity and angular displacement information of the projectile rotation. The cumulative error during the measurement is eliminated by using the periodically pulse clear method, thereby realizing the measurement of the angular motion parameters of the high-speed rotating projectile, and satisfying its measurement requirements of wide range of rotational speed and high precision. The proposed method has definite value to engineering applications in the fields of attitude determination and geomagnetic navigation [16].

## 2. Measurement Principle of the Sensor

### 2.1. Basic Principle of Measuring Angular Velocity

**Figure 1:**
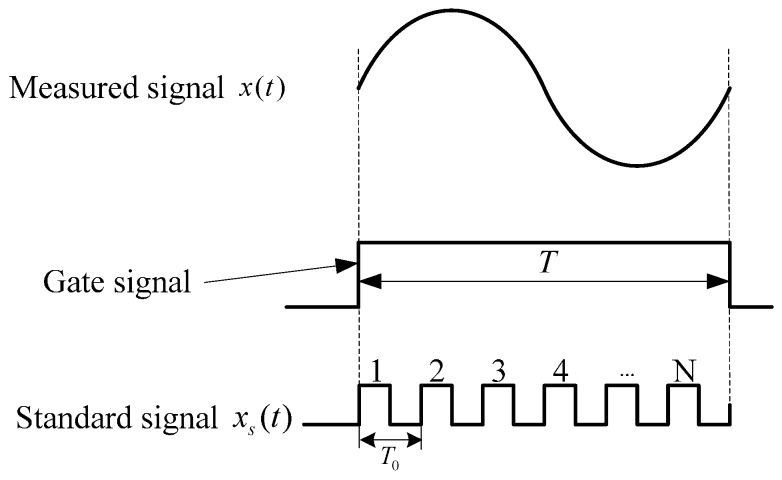
Schematic diagram of frequency measurement based on period method.

The measurement method of angular velocity proposed in this paper draws from the theory of digital frequency meters. The basic principle is shown in Figure 1.

A measured signal *x*(*t*) is converted into a gate signal through a shaping circuit. Under the circumstances that the standard frequency signal *x_s_*(*t*) with higher frequency is taken as a periodic pulse signal, the measured signal is calculated by pulse counting within a gate time *T*. Assuming that the count value is *N*, the cycle of measured signal and standard signal are *T_x_* and *T*_0_, respectively. Then, the cycle of the measured signal is:

(1) $$T_{x}=N*T_{0}$$

and the frequency of the measured signal is:

(2) $$f_{x}=\frac{f_{0}}{N}$$

It is known by observation of Equations (1) and (2) that when counting a standard frequency signal with periodic pulse counting, the frequency of the measured signal can be calculated accordingly. However, rotational angular motion parameters of the projectile measured in this paper include not only the angular velocity information of the projectile rotation, but also real-time angular displacement information of the rotation. For the angular displacement information, the rotation angle of projectile that one cycle *T_x_* of the measured signal corresponds to 360°. The angular displacement is output in a cumulative way by using standard frequency signal *x_s_*(*t*). The period of each pulse represents a fixed step-progressive accumulation angle *θ*, where *θ* = 360°/*N*. In this way, *θ* accumulates to 360° with *N* cycles. Because the angular displacement that each pulse represents is fixed, therefore, the count value *N* is a stable constant. Variable *N* stands for the resolution of the angular displacement. However, in the process of frequency measurement, the frequency of a standard signal is changeless, while the count value *N* varies with the difference of the measured signal. Consequently, angular displacement information cannot be output effectively. Aiming at the problems mentioned above, this paper presents a self-adaptive frequency tracking measurement algorithm. On the premise that the value of pulse counting is *N*, the algorithm realizes measurement of angular displacement and angular velocity by changing the frequency of the standard signal self-adaptively.

### 2.2. Principle of Self-Adaptive Frequency Tracking Measurement

**Figure 2:**
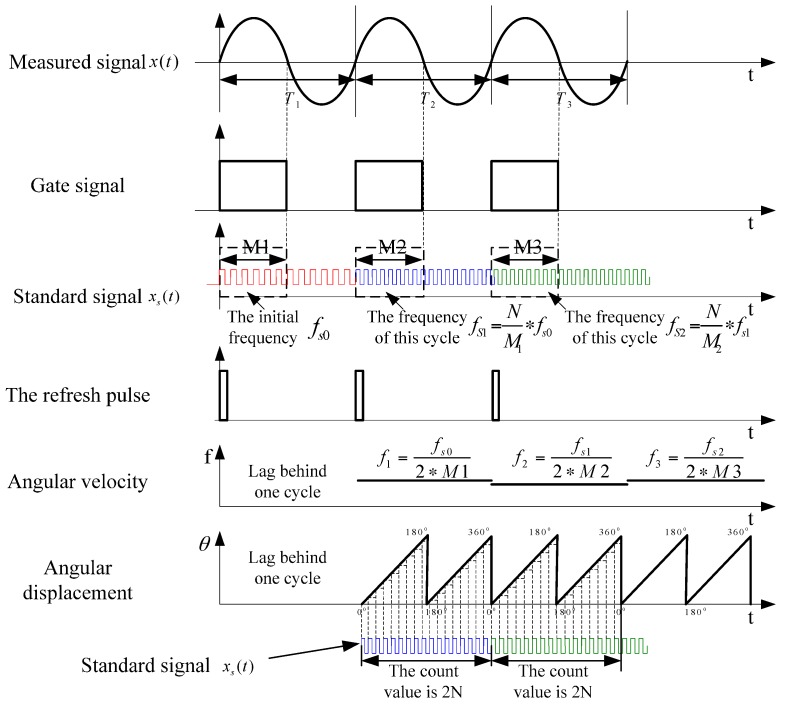
Schematic diagram of the self-adaptive frequency tracking measurement method.

The principle of the self-adaptive frequency tracking measurement method is shown in Figure 2. Supposing that measured signal is *x*(*t*), the standard signal is *x_s_*(*t*). When the pulse counting value is fixed at *N*, the cumulatively output angular displacement is 180°. The frequencies of the first three cycles of the measured signal are *f*_1_, *f*_2_ and *f*_3_, respectively. The corresponding frequencies of the first three cycles of the standard signal are *fs*_0_, *fs*_1_ and *fs*_2_, respectively. The half period of the measured signal *x*(*t*) is taken as gate time after processing the signal with shaping circuit modulation.

Presupposing that the initial frequency of standard signal is *fs*_0_, the count value is *M*_1_ during the gate time of the first cycle of the measured signal. Then, the current angular velocity can be calculated by using Equation (3):

(3) $$f_{1}=\frac{1}{2*M_{1}}*f_{s0}$$

In order that the angular displacement can be output effectively after calculating the angular velocity, when requiring that the value of pulse counting be *N* within the same time period, the frequency of the standard signal *x_s_*(*t*) should be updated by using Equation (4):

(4) $$f_{s1}=\frac{N}{M_{1}}*f_{s0}$$

Since the count value of standard signal is *M*_1_ under the current cycle, the angular displacement information cannot be effectively output yet. From the second cycle on, the detailed process is as follows:

(1) Updating the frequency of standard signal as *fs*_1_, outputting angular displacement information of the first cycle in a cumulative way. Two sawtooth waves (STWs) are output in one cycle. The value of pulse counting that each STW corresponds to is *N*. The first STW corresponds to an angular displacement from 0° to 180°, and the second STW corresponds to an angular displacement from 180° to 360°.
(2) Outputting angular velocity *f*_1_ of the first cycle.
(3) Measuring angular velocity of the current cycle by utilizing standard signal *Js*_1_ and Equation (3), and updating the frequency of standard signal by using Equation (4).

In this way of combining step (1), (2) with (3), the frequency of the standard signal can be updated self-adaptively, and the angular velocity and angular displacement information can be output with a mode which lags one cycle behind the measured signal. Simultaneously, the designed system outputs the refresh pulse, which represents the initial zero phase of every cycle of the measured signal. The refresh pulse can provide input attitude parameters to a subsequent inertial navigation system (INS) by cooperating with angular displacement information.

## 3. Design of Angular Velocity Sensor

**Figure 3:**
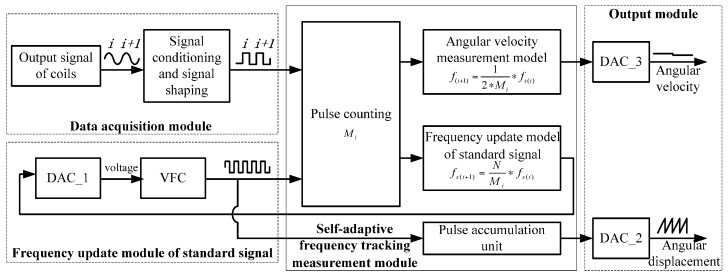
Schematic diagram of the general design scheme of the sensor.

System architecture and a specific design scheme of the prototype of the sensor are given in Figure 3. The sensor consists of five parts, including a data acquisition module, standard signal frequency update module, self-adaptive frequency tracking measurement module and output module.

### 3.1. Data Acquisition Module

**Figure 4:**
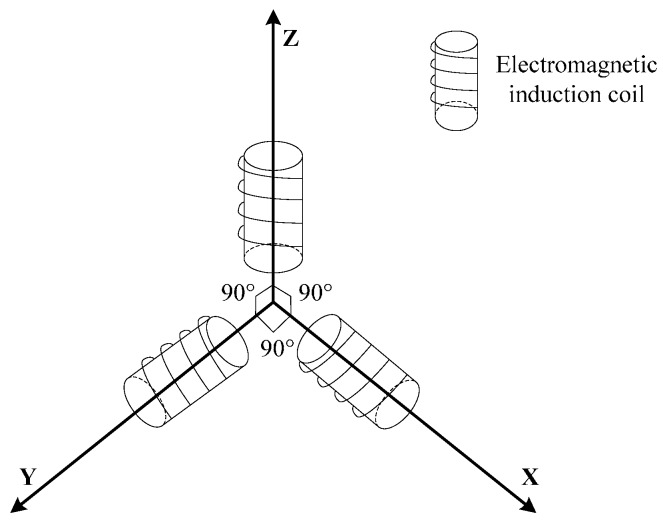
Schematic diagram of installation method of triaxial coils.

Under the conditions of different rotational speeds of a high-speed rotating projectile, an electromagnetic induction coil is employed to obtain the real-time information about the projectile movement across a geomagnetic field. According to Faraday’s Law of Electromagnetic Induction, a framework of a bent “I-shape” type is used to wind an uniaxial coil, whose wire diameter is 0.09 mm, number of turns is 9500, and resistance is about 665 Ω. Simultaneously, triaxial coils are installed in a mutually orthogonal way to realize the measurement of triaxial geomagnetic signals, as shown in Figure 4.

The relationships between the test frequency and output voltage of the wound field coil are listed in Table 1.

**Figure 5:**
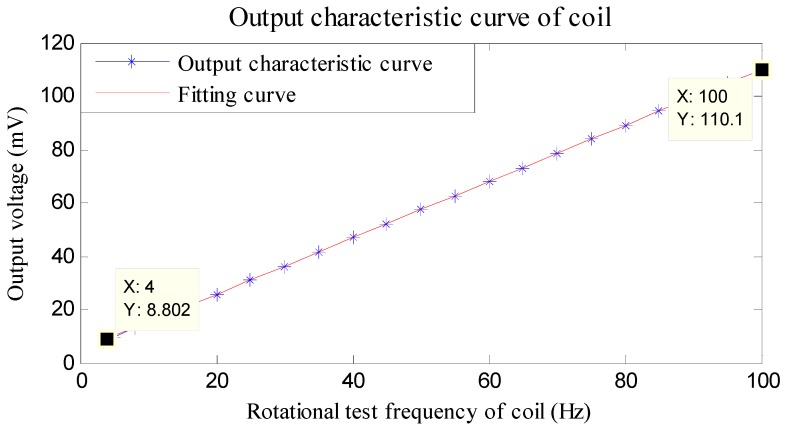
Output characteristic curve of coil.

The linearity of the induction coil is calibrated by linear fitting. The characteristic output curve and fitting curve are shown in Figure 5.

Figure 5 reveals that when employing an induction coil to cut a fixed magnetic field, the frequency of the induced magnetic signal is in a range from 4 Hz to 100 Hz. The cutting frequency and induced voltage generated show a good linear relationship with linearity of 0.5% F.S., thereby satisfying the measurement requirements of a large range of rotational speeds for a high-speed rotating projectile. Weak signals induced by the coils are amplified self-adaptively within an appropriate range by using a signal conditioning circuit, so as to output gate signals after half-wave shaping.

### 3.2. Self-Adaptive Frequency Tracking Measurement Module and Frequency Update Module

A digital to analog converter (DAC) is utilized to control voltage-frequency converter (VFC) and make it output a standard signal *x_s_*(*t*). The frequency of the standard signal is used to update Equation (4). The standard signal is updated under the control of the VFC at the initial time of every cycle of the measured signal.

The relationship between DAC and VFC is as follows. Assuming that output voltage of DAC is *V_i_* in the *i*-th cycle, the corresponding output frequency of VFC is *f_si_*. According to the transmission characteristics of the VFC, the connection between these two parameters above is:

(5) $$f_{si}=a*V_{i}+b$$

where *a* and *b* are linear constants of the VFC obtained through a multi-point fitting method.

Combined with Equation (4), the corresponding output voltage of DAC of the next cycle is:

(6) $$V_{i+1}=\frac{N}{M_{i}}*V_{i}+\frac{b}{a}*\frac{\left(N-M_{i}\right)}{M_{i}}$$

Based on the approach that an angular velocity measurement model based on Equation (6) is built by taking advantage of FPGA, the value of DAC can be updated. Simultaneously, the count value *M_i_* is latched up, and then the angular velocity can be calculated by using Equation (3).

### 3.3. Output Module

The angular velocity and angular displacement information are both output in the form of voltage by utilizing the DAC. The angular displacement is output in the form of STW. Two STW are output in one cycle. When the pulse counting value reaches *N*, the DAC outputs a STW signal with presupposed amplitude. The full voltage of each STW corresponds to angular displacements of 180° and 360°, respectively. The corresponding angular displacement can be calculated by using the output voltage.

## 4. Experimental Verification

### 4.1. Linearity Calibration of the VFC

The sensitivity of the VFC determines the corresponding relation between the regulating voltage of the DAC and VFC. In order to realize a self-adaptive frequency tracking measurement algorithm, the sensitivity of the VFC is calibrated before experiments. An AD654 of Analog Device Instrument (ADI Company, Norwood, MA, USA) is chosen as VFC, whose relationships between input voltage and output frequency are listed in Table 2.

**Figure 6:**
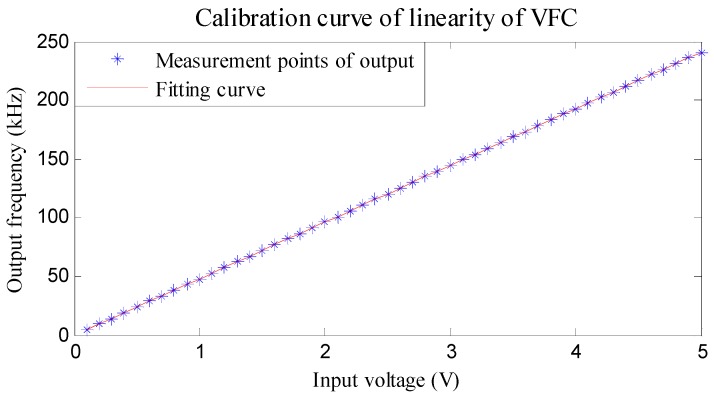
Calibration curve of linearity of VFC.

The calibration curve of the VFC’s linearity is obtained according to Table 2, as shown in Figure 6.

Based on the linear fitting approach, the corresponding output characteristic equation of the VFC, which also implies the relationship between regulating voltage of the DAC and VFC is expressed as:

(7) f=48.2499∗V−0.3473

It can be concluded that the linearity of the selected VFC is 0.052% F.S. This good linearity can satisfy the high precision demands for measuring the angular velocity of high-speed rotating projectiles.

### 4.2. Semi-Physical Simulation Experiment—Alternating Magnetic Field

**Figure 7:**
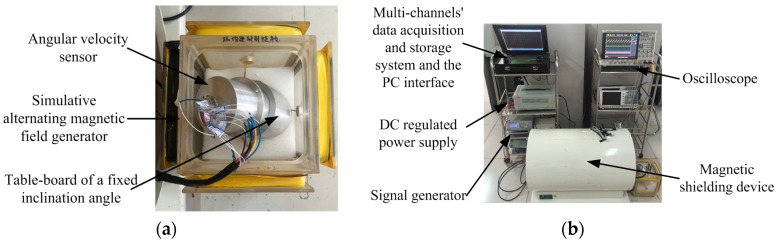
Experimental test platform of the simulative alternating magnetic field: (**a**) Simulative magnetic field generator; (**b**) Experimental test platform.

In order to calibrate the angular velocity measurement accuracy of the sensor, simulation experiments are carried out on the basis of using a magnetic shielding device and simulative alternating magnetic field generator of Helmholtz coils. The novel sensor researched and developed based on the designed system is gently put in a simulative alternating magnetic field of Helmholtz coils as shown in Figure 7. The inclination angle of the fixed table-board is set as 30° to ensure that under the action of the alternating magnetic field, the triaxial electromagnetic induction coils can all output cutting magnetic induction signals. Furthermore, the frequency of the signal generator, which represents the rotational speed frequency of a projectile under actual working conditions, is set at a range from 5 Hz to 100 Hz. The intensity of the applied magnetic field is ±1 Gauss. The count pulse value is 1024 per half cycle, which means that *N* = 1024.

**Figure 8:**
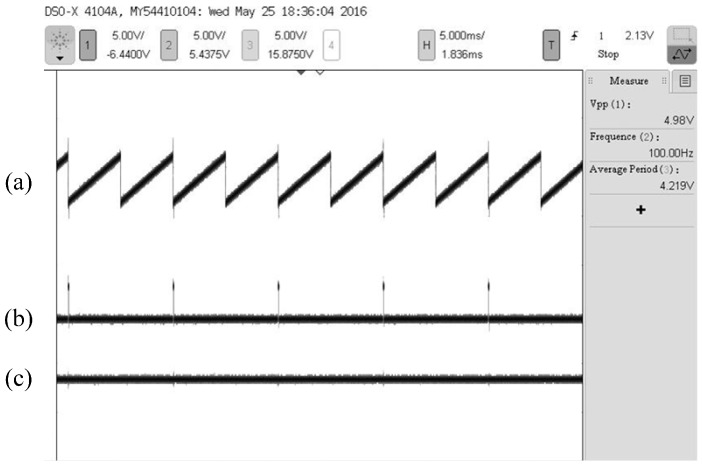
Oscillogram of the sensor output signal with a simulative rotational speed of 100 rps: (**a**) Angular displacement information; (**b**) The refresh pulse; (**c**) Angular velocity information.

Under the condition that the frequency of the alternating magnetic field is 100 Hz, the oscillogram of the output signal of the sensor is shown in Figure 8.

As can be seen from Figure 8, there are three types of signal output by the sensor: angular velocity signal, angular displacement signal and the refresh pulse signal. When the presupposed frequency of the alternating magnetic field is 100 Hz, the angular velocity signal is stable with small ripples of less than 40 mV. The corresponding frequency of the refresh pulse stabilizes at 100 Hz, which illustrates that the designed sensor can accurately detect the initial zero during every cycle and achieve the function of automatic zero clearing during the process of measurement. Simultaneously, two STW representing angular displacement information from 0° to 360° are output between two refresh pulses. Experiments are conducted with the preset values listed in Table 3 one after another. Under different frequencies, the corresponding angular velocity parameter information and measurement errors are acquired as follows.

As Table 3 shows, along with the change of alternating frequency of the magnetic field, the sensor can realize angular velocity measurements within an extremely high dynamic range from 5 Hz to 100 Hz, with a maximum measurement error of less than 0.3%.

### 4.3. Physical Simulation Experiment—Three-Axis Flight Turntable

**Figure 9:**
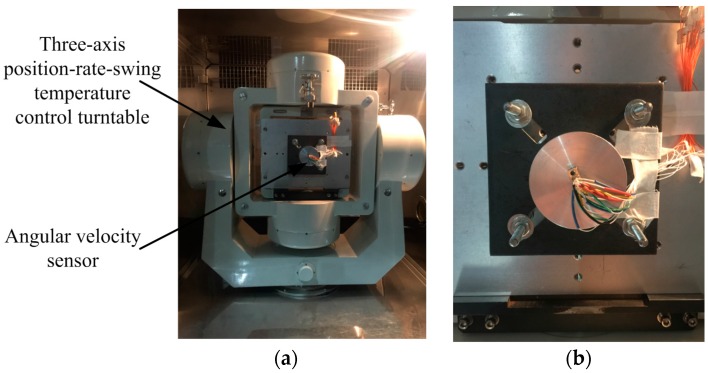
Experimental three-axis flight turntable test platform: (**a**) Three-axis turntable; (**b**) Fixed platform of the sensor.

In order to calibrate the angular displacement measurement accuracy of the sensor, test experiments were conducted using a three-axis position-rate-swing temperature control flight turntable. The highest test frequency of the turntable is 1000°/s, with a rate precision error of ±0.01°. The angular velocity sensor is fixed at the heart of the turntable, as shown in Figure 9. The turntable is controlled and adjusted to skew 30°. The rotational test frequency of the inner frame of the turntable is set at different angular rotation frequencies.

**Figure 10:**
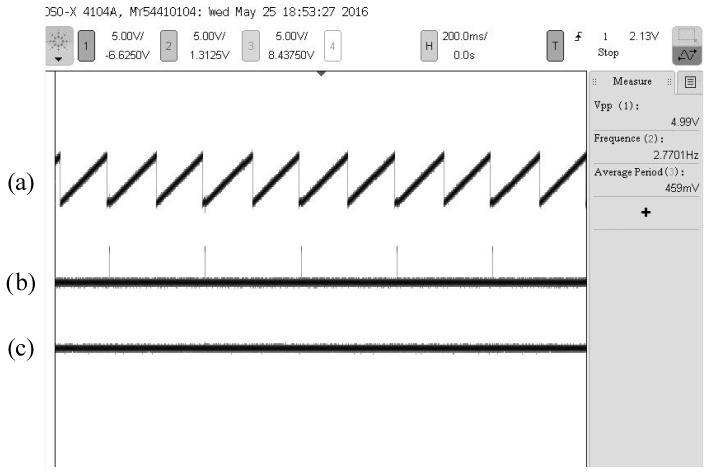
Oscillogram of the sensor output signal with a simulative rotational speed of 1000°/s: (**a**) Angular displacement information; (**b**) The refresh pulse; (**c**) Angular velocity information.

Under the condition that the rotational test frequency of the inner frame of the turntable is 1000°/s, an oscillogram of the sensor output signal is shown in Figure 10.

It can be seen from Figure 10 that when rotational test frequency of the turntable is 1000°/s, the corresponding frequency of the refresh pulse stabilizes at 2.770 Hz, which shows that the sensor can accurately detect the initial zero during every cycle and achieve the function of automatic zero clearing during the measurement. Simultaneously, the output STW of the sensor is stable, the initial zero of the STW and the refresh pulse have a one-to-one correspondence. The output DC voltage of the regulation DAC is steady with small ripples, which illustrates that the sensor has stable performance, and the obtained angular displacement is accurate.

**Figure 11:**
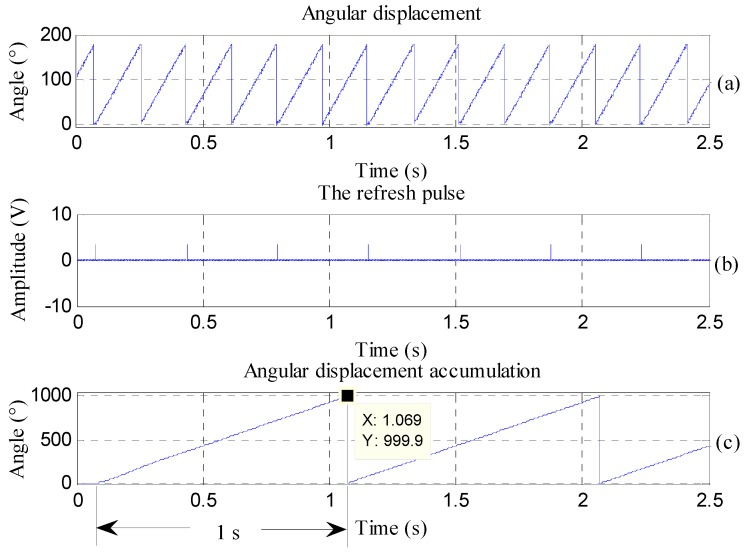
Accumulative calculation graph of angular displacement at 1000°/s.

The voltage information of the angular displacement signal, obtained through Figure 10, is converted into angle information (as shown in Figure 11a) by integrating with a time horizon of 1 s (as shown in Figure 11c). The rotational frequency of the turntable is 1000°/s, which means that the angular displacement accumulates 1000° in 1 s. When integrating the actual angular displacement signal, the error is 0.1°, thus satisfying the measurement requirements.

Multi-group experiments are carried out according to presupposed rotation frequencies. The corresponding experimental test results are given in Table 4.

On the basis of the analysis of Table 4, it is concluded that when the inner frame of the turntable rotates at 600, 900 and 1000°/s, respectively, the measurement errors of the angular displacement acquired through the sensor are all less than 0.2°, thereby satisfying the actual angular displacement measurement requirements for a high-speed rotating projectile.

## 5. Conclusions

Aiming at meeting the demands for measuring angular motion parameters of high-speed rotating projectiles, this paper puts forth a new method for measuring the angular velocity and angular displacement parameters of the rotation of a projectile based on the electromagnetic induction principle, and also designs the corresponding sensor. To begin with, triaxial electromagnetic induction coils are used to acquire the cutting geomagnetic signals of the projectile. Next, a self-adaptive frequency tracking measurement algorithm, which combines pulse counting with a way of updating the frequency of a standard signal self-adaptively, is proposed by utilizing DAC and VFC to calculate the angular velocity and angular displacement information, and a principle prototype of the sensor is designed based on FPGA. Third, angular velocity performance tests are carried out by using a simulative alternating magnetic field generator of Helmholtz coils, within the alternative frequency range from 5 rps to 100 rps. The measurement errors are all less than 0.3%. Finally, angular displacement performance tests are conducted by utilizing a three-axis position-rate-swing temperature control turntable, in a range from 1 rps to 3 rps. The angular displacement measurement errors are all lower than 0.2°. The experimental results both show that the sensor can measure angular motion information within a range from 1 rps to 100 rps, and can realize real-time measurements of the angular motion parameters of a high-speed rotating projectile with high precision.

Two research topics are scheduled to be developed in the near future. On the one hand, the final purpose of the sensor is to provide motion parameters for attitude determination of high-speed rotating projectiles and geomagnetic navigation. How to construct a navigation model and match with the interface of the current navigation system would be a direction for further research. On the other hand, the stability and balance of the self-adaptive frequency tracking measurement model should be analyzed to apply the available sensor for whole trajectory measurement of high-speed rotating projectiles.

## Figures and Tables

**Table 1:** Relationship between frequency and voltage of output signals of coil (Frequency: Hz; Output voltage: mV).

Frequency	Output Voltage	Frequency	Output Voltage	Frequency	Output Voltage
4	8.30	45	52.10	75	83.70
15	20.50	50	57.40	85	94.30
25	31.00	55	62.70	95	104.80
35	41.60	65	73.20	100	110.00

**Table 2:** Calibration table of VFC (Voltage: V; Frequency: kHz).

Voltage	Frequency	Voltage	Frequency	Voltage	Frequency	Voltage	Frequency	Voltage	Frequency
0.1	4.48	1.1	52.72	2.1	100.97	3.1	149.20	4.1	197.66
0.3	14.20	1.3	62.30	2.3	110.42	3.3	158.70	4.3	207.26
0.5	23.65	1.5	71.97	2.5	120.25	3.5	168.75	4.5	216.94
0.7	33.57	1.7	81.50	2.7	129.70	3.7	178.21	4.7	226.78
0.9	43.17	1.9	91.26	2.9	139.50	3.9	187.91	4.9	236.21

**Table 3:** Measurement results of angular velocity parameter.

Serial Number	Presupposed Frequency/Hz	Voltage Corresponding to Angular Velocity/V	Angular Velocity/rps	Measurement Error/%	RMS Noise/mV
1	5	0.219	4.9851	0.298	36
2	10	0.430	9.9709	0.291	35
3	20	0.854	19.9430	0.285	32
4	30	1.277	29.9159	0.280	35
5	50	2.124	49.8708	0.258	30
6	70	2.971	69.8258	0.249	32
7	90	3.818	89.7807	0.244	34
8	100	4.219	99.7699	0.230	30

RMS noise: the root-mean-square value of noise.

**Table 4:** Experimental test results of three-axis flight turntable.

Serial Number	Rotational Frequency of Three-Axis Turntable/°/s	Angular Velocity/rps	Angular Displacement/°/s	Measurement Error/°
1	600	1.6667	599.8	0.2
2	900	2.5020	900.2	0.2
3	1000	2.7770	999.9	0.1
